# Molecular Characterization of Hemotropic *Mycoplasma* spp. From Bats (Chiroptera) in the Northern Pantanal, Brazil

**DOI:** 10.3390/pathogens15060654

**Published:** 2026-06-22

**Authors:** Nathalia de Assis Pereira, Juliane Saldanha, Thiago Borges Fernandes Semedo, Guilherme Siniciato Terra Garbino, Rogério Vieira Rossi, Sofia de Souza Pereira Gomes, Sayanne Luns Hatum, Thállitha Samih Wischral Jayme Vieira, Rafael Felipe da Costa Vieira, Jansen de Araújo, Edison Luiz Durigon, Daniel Moura de Aguiar

**Affiliations:** 1 Laboratory of Virology and Rickettsiosis, Faculty of Veterinary Medicine, Federal University of Mato Grosso State, Cuiabá 78060-900, Brazil; nathaliaassis89@gmail.com (N.d.A.P.); jusaldanhasilva@gmail.com (J.S.); thiagosemedo@gmail.com (T.B.F.S.); sofiasouzapg@gmail.com (S.d.S.P.G.); sayhatum@gmail.com (S.L.H.); 2 CIBIO, Centro de Investigacão em Biodiversidade e Recursos Genéticos, Universidade do Porto, 4099-002 Porto, Portugal; 3 Departamento de Biologia, Universidade do Porto, 4099-002 Porto, Portugal; 4 BIOPOLIS Program in Genomics, Biodiversity and Land Planning, 4099-002 Porto, Portugal; 5 Museu de Zoologia João Moojen, Departamento de Biologia Animal, Universidade Federal de Vicosa, Viçosa 36570-900, Brazil; guilherme.garbino@ufv.br; 6 Laboratory of Mastozoology, BioScience Institute, Federal University of Mato Grosso State, Cuiabá 78060-900, Brazil; rogerrossi@gmail.com; 7 Department of Chemistry, The University of North Carolina at Charlotte, Charlotte, NC 28223, USA; t.vieira@charlotte.edu; 8 Department of Epidemiology and Community Health, The University of North Carolina at Charlotte, Charlotte, NC 28223, USA; rvieira@charlotte.edu; 9 Center for Computational Intelligence to Predict Environment and Health Risks, The University of North Carolina at Charlotte, Charlotte, NC 28223, USA; 10 Emerging Viruses Research and Clinical and Molecular Virology Laboratories, Institute of Biomedical Sciences, University of São Paulo, São Paulo 05508-000, Brazil; jansentequila@usp.br (J.d.A.); eldurigo@usp.br (E.L.D.); 11 Institut Pasteur of São Paulo, São Paulo 05508-020, Brazil

**Keywords:** mammals, hemoplasmas, *Molossops temminckii*, PCR, detection

## Abstract

In this study, we conducted a molecular investigation of hemotropic *Mycoplasma* spp. in bat species captured in the northern region of the Pantanal biome, Mato Grosso State, Brazil. Tissue samples were screened by qPCR targeting the 16S rRNA gene. Positive samples were subsequently subjected to conventional PCR assays targeting partial fragments of the 16S rRNA (~900 bp) and 23S rRNA (~800 bp) genes. Hemoplasma DNA was detected in four bat species: *Glossophaga soricina*, *Molossops temminckii*, *Molossus rufus*, and *Desmodus rotundus*. Phylogenetic analyses based on partial 16S and 23S rRNA gene sequences demonstrated that the detected hemoplasmas clustered predominantly with previously described bat-associated hemoplasmas from Brazil and other countries in the Americas. Notably, the detection in *M. temminckii* represents, to our knowledge, the first molecular evidence of hemotropic Mycoplasma infection in this bat species. These findings expand current knowledge regarding the occurrence, host range, and genetic diversity of hemotropic *Mycoplasma* spp. in bats from the Pantanal biome and contribute to wildlife surveillance efforts in this ecologically important region.

## 1. Introduction

Bats (order Chiroptera) are the second most species-rich group of mammals in the tropics [1]. This diversity is associated with a wide global distribution, excluding only polar regions, extreme desert climates, and some oceanic islands [2]. Some environmental factors limit the occurrence and abundance of some species, including habitat heterogeneity and seasonality [3]. Mato Grosso is a state located in the Central-West region of Brazil. With an area approximately the size of France, it harbors extensive forested habitats including the Amazon, Cerrado (savanna), and Pantanal biomes. In terms of bat diversity, approximately 99 bat species are known in Mato Grosso [4].

Beyond their ecological importance, bat communities from the Pantanal harbor a wide diversity of microorganisms and may contribute to the maintenance of host-associated microbial diversity in natural ecosystems, particularly in areas undergoing environmental and anthropogenic changes [5].

Bats harbor a wide diversity of pathogens, including viruses, bacteria, protozoa, and fungi, and exhibit remarkable resistance to a range of infections [2]. Hemoplasmas, particularly those belonging to the hemotropic Mycoplasma group, have attracted increasing attention due to their wide distribution among domestic and wild mammals and their genetic diversity in different host species [6]. Hemotropic *Mycoplasma* spp. (hemoplasmas), members of the genus *Mycoplasma* within the order *Mycoplasmatales* and family *Mycoplasmataceae*, are wall-less bacteria that parasitize erythrocytes and may cause manifestations ranging from asymptomatic infections to chronic disease in mammalian hosts [7]. Notably, hemoplasmas can induce severe hemolytic anemia by adhering to erythrocyte surfaces, leading to indentation or deformation of the host cell membrane [8]. The severity of the disease depends on the genotype or involved species [9]. In Brazil, hemoplasma infections have been reported in wild mammals such as canids, felids, primates, and in bats, including hematophagous species [10,11,12,13,14]. Although the pathogenic and zoonotic significance of most bat-associated hemoplasmas remains poorly understood, *Candidatus* Mycoplasma haematohominis has been reported in both bats and humans from French Polynesia (Melanesia), highlighting the importance of continued surveillance and molecular characterization of bat-associated hemoplasmas in wildlife populations [15].

Hematophagous arthropods, such as fleas and ticks, have been proposed as potential vectors of some *Mycoplasma* species. However, vertebrate hosts can also be infected in other ways, including subcutaneous, intravenous, intraperitoneal, and oral inoculation with contaminated blood [12]. Metagenomic analyses have also demonstrated the presence of hemoplasmas in the saliva of vampire bats (*Desmodus rotundus*), suggesting the possibility of direct transmission through bites from these animals [16].

Studies involving the molecular characterization of hemoplasmas are important for improving our understanding of their genetic diversity, host associations, and geographic distribution in natural ecosystems [17]. Such information contributes to wildlife surveillance efforts and expands current knowledge regarding host-associated microorganisms in biodiversity-rich environments [17]. In this context, considering the rich bat diversity of the Pantanal and the limited information available on hemoplasmas in this biome, this study aimed to investigate the presence and genetic diversity of hemotropic *Mycoplasma* spp. in bats sampled from different areas of the Pantanal biome in Mato Grosso State, Brazil. By combining molecular detection and phylogenetic analyses based on partial 16S and 23S rRNA gene sequences, this study contributes to current knowledge of hemoplasma diversity in bats from a poorly studied wetland ecosystem.

## 2. Materials and Methods

**Figure 1:**
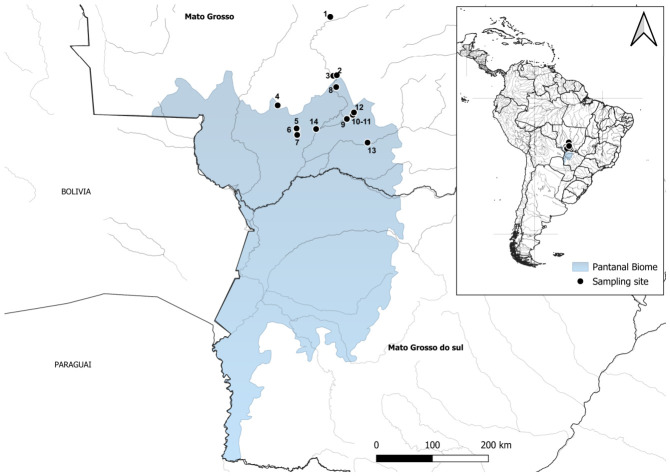
The geographic location of bat collecting sites in the Pantanal and Cerrado Biomes of Mato Grosso state, Brazil. Geographic coordinates of bat collection sites, their municipalities and localities in the Pantanal and Cerrado biomes are showed in the Table S1.

This study was conducted in the municipalities of Cuiabá, Nobres, Poconé, and Santo Antônio do Leverger, in the state of Mato Grosso, Brazil, from February to August 2021, as described in Magalhães et al., 2025 [5]. These sampling sites were selected because they represent different environments and are relatively close to urban centers or local communities (Figure 1).

The collection methods used to capture the bats included active searches aided by hand nets and mist nets. Bats were identified using taxonomic keys [18]. After identification, the collected bats were euthanized in accordance with the guidelines of the regional Veterinary Medicine Council of Mato Grosso state [19]. Samples of lung and spleen were collected and stored in cryotubes at −80 °C. DNA extraction was performed with 20 mg of tissue using a commercial kit (MagMAX™ CORE nucleic acid purification kit, Applied Biosystems™, San Francisco, CA, USA) following the manufacturer’s instructions. A conventional PCR assay, targeting a fragment of the mammalian endogenous gene glyceraldehyde-3-phosphate dehydrogenase (*gapdh*) [20], was performed on all samples to monitor DNA extraction.

Bat samples were screened using a universal SYBR Green real-time PCR (qPCR) assay targeting the 16S rRNA gene of hemoplasmas [21]. Reactions were performed in a final volume of 10 µL containing PowerTrack™ SYBR Green Master Mix (Applied Biosystems™, Thermo Fisher Scientific, Waltham, MA, USA) and primers at a final concentration of 0.5 µM each, following the manufacturer’s recommendations. Amplifications were performed with annealing/extension at 60 °C for 1 min. Melting curve analysis consisted of 95 °C for 15 s and 60 °C for 1 min, both at a ramp rate of 1.6 °C/s, followed by a dissociation step at 95 °C for 15 s at a ramp rate of 0.05 °C/s. Samples with Ct values < 38 were considered positive. A previously confirmed hemoplasma-positive canine sample was used as positive control, while nuclease-free water was included as negative control. All reactions were performed in duplicate.

Positive samples detected by qPCR were further analyzed by conventional PCR assays using genus-specific primers targeting partial fragments of approximately 900 bp of the 16S rRNA gene [22] and 800 bp of the 23S rRNA gene [23]. Conventional PCR reactions were performed in a final volume of 50 µL using GoTaq^®^ G2 Colorless Master Mix (Promega Corporation, Madison, WI, USA). Amplifications consisted of 35 cycles with annealing at 55 °C for 30 s and extension at 72 °C for 1 min.

PCR products were visualized by electrophoresis on 1% agarose gels stained with GelRed™ Nucleic Acid Gel Stain (Biotium, Fremont, CA, USA) and examined using a ChemiDoc XRS system (Bio-Rad, Hercules, CA, USA). Amplicons of the expected size were purified using a commercial purification kit (ReliaPrep™ DNA Clean-Up and Concentration System, Promega Corporation, Madison, WI, USA) and subjected to bidirectional Sanger sequencing using the same primers employed in the PCR assays.

Raw forward and reverse chromatograms were manually inspected in Geneious Prime^®^ (version 2024.0.7, Biomatters Ltd., Auckland, New Zealand). Low-quality regions at the beginning and end of each read were trimmed based on chromatogram quality, retaining only regions with clearly resolved peaks for downstream analyses. Forward and reverse reads were assembled to generate consensus sequences, and ambiguous positions were checked manually and resolved when supported by chromatogram inspection.

The generated 16S and 23S rRNA gene sequences were processed and analyzed using Geneious Prime^®^. The sequences were subsequently compared with the DNA database using the BLAST^®^ algorithm (version 2.8.0) from the National Center for Biotechnology Information (NCBI) (http://www.ncbi.nlm.nih.gov/BLAST/Blast.cgi; accessed on 20 April 2026) to determine the closest identities to generic organisms available in GenBank^®^.

The nucleotides sequences of 16S from two samples and 23S rRNA from four samples were aligned using MAFFT [24] and subsequently compared to each other and to 28 reference sequences retrieved from GenBank (Table S2). Phylogenetic analyses were performed separately for the partial 16S and 23S rRNA gene datasets using the Maximum Likelihood (ML) method implemented in MEGA version 12.1. The best-fit nucleotide substitution models were selected independently for each dataset according to the Bayesian Information Criterion (BIC). The evolutionary history of the 16S rRNA dataset was inferred using the Tamura 3-parameter model with Gamma distribution and invariant sites (T92+G+I) [25], whereas the 23S rRNA dataset was reconstructed using the Tamura 3-parameter model with Gamma distribution (T92+G) [25]. Bootstrap support was assessed using 1000 replicates. Partial deletion with a 90% site coverage cutoff was applied to eliminate positions containing excessive gaps or missing data. The 16S and 23S rRNA trees included *Ureaplasma parvum* (PV081195 and NR_076563, respectively) as outgroups.

## 3. Results

In total, 157 bats of 26 species were sampled belonging to the following families: Phyllostomidae, Molossidae, Noctilionidae, Vespertilionidae, Mormoopidae, and Emballonuridae. The Phyllostomidae family had the largest number of captures (105), including *Carollia perspicillata* (18), *Glossophaga soricina* (45), *Platyrrhinus lineatus* (18), *D. rotundus* (13) and *Macrophyllum macrophyllum* (1). A single Noctilionidae species, *Noctilio albiventris* (11), was also commonly captured. Two bat specimens could not be identified to the species level; the following genera were identified: *Lophostoma* (1) and *Pteronotus* (1). From the total sampled bats, 87 were females, 68 were males, and two samples were without sex determination. In terms of trophic guilds, nectarivorous bats accounted for 45 specimens, frugivorous for 42 specimens, insectivorous for 31, omnivores for 26, and 13 specimens were blood-feeding bats.

Bats were sampled in the municipalities of Cuiabá (n = 4), Nobres (n = 9), Poconé (n = 40) and Santo Antônio de Leverger (n = 104). Active search using hand nets accounted for 68.2% (n = 107) of the captures, while 31.8% (n = 50) were collected using mist nets.

Molecular detection of hemoplasmas was observed in five (3.18%) samples. Positive bats were *G. soricina*, *Molossus rufus*, *D. rotundus,* and *Molossops temminckii*. Out of the five samples, two were positive for the 16S gene and four for 23S. Table 1 shows additional data for the positive bats, including their GenBank^®^ accession numbers.

Sequences of the 16S rRNA gene showed 94.0% similarity among them, whereas similarities with sequences deposited in GenBank ranged from 99.03% to 99.68%. Likewise, sequences of the 23S rRNA gene showed 91.0–100% similarity among themselves and 99.00–99.83% similarity with sequences available in GenBank. Detailed BLAST results for all sequences generated in the present study are provided in Table S3.

**Figure 2:**
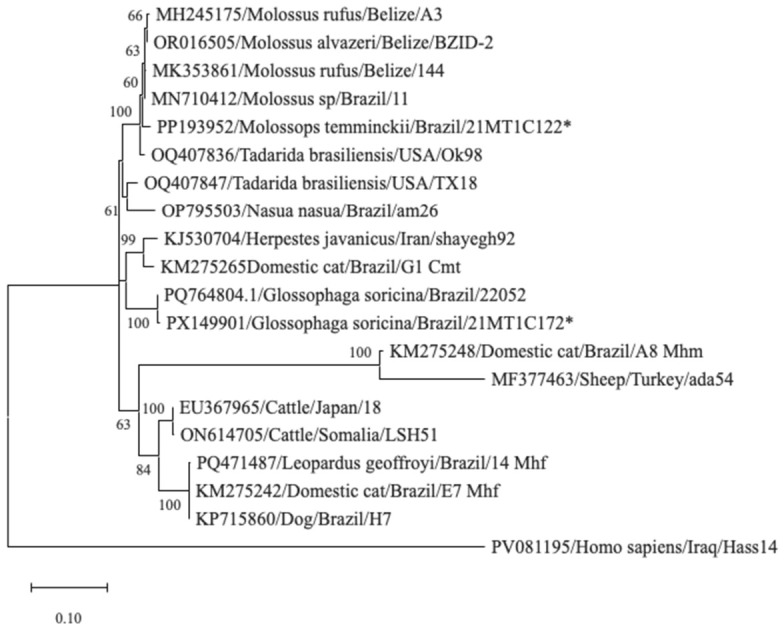
Maximum Likelihood phylogenetic tree based on partial 16S rRNA gene sequences of hemotropic *Mycoplasma* spp. detected in bats from the Pantanal, Brazil. The analysis included 20 sequences and 626 aligned nucleotide positions. The T92+G+I substitution model was selected according to the Bayesian Information Criterion (BIC). Bootstrap values ≥60% (1000 replicates) are shown at the nodes. Sequences generated in the present study are indicated by an asterisk.

Phylogenetic analysis based on partial 16S rRNA gene sequences demonstrated that the hemotropic *Mycoplasma* spp. sequence detected in *M. temminckii* (PP193952) clustered with sequences previously identified in bats from Belize and Brazil, including *M. rufus*, *Molossus alvarezi*, and *Molossus* sp. (Figure 2). The sequence obtained from *G. soricina* (PX149901) clustered with another hemotropic *Mycoplasma* spp. sequence previously detected in *G. soricina* from Brazil, forming a well-supported clade (bootstrap = 100). Additional related sequences included hemoplasmas previously reported in *Herpestes javanicus* from Iran and domestic cats from Brazil.

**Figure 3:**
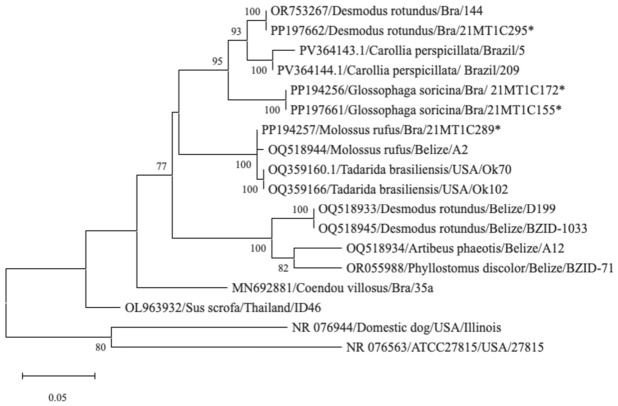
Maximum Likelihood phylogenetic tree based on partial 23S rRNA gene sequences of hemotropic *Mycoplasma* spp. detected in bats from the Pantanal, Brazil. The analysis included 18 sequences and 452 aligned nucleotide positions. The T92+G substitution model was selected according to the Bayesian Information Criterion (BIC). Bootstrap values ≥70% (1000 replicates) are shown at the nodes. Sequences generated in the present study are indicated by an asterisk.

Phylogenetic analysis based on partial 23S rRNA gene sequences demonstrated that the hemotropic *Mycoplasma* spp. sequences detected in bats from the Pantanal biome clustered with previously described bat-associated hemoplasmas from Brazil, Belize, and the United States (Figure 3). The sequence obtained from *D. rotundus* (PP197662) clustered with a hemotropic *Mycoplasma* spp. previously detected in *D. rotundus* from Brazil, forming a strongly supported clade (bootstrap = 100). Likewise, the sequences detected in *G. soricina* (PP194256 and PP197661) formed a well-supported cluster associated with hemoplasmas previously reported in neotropic phyllostomid bats (bootstrap = 100). The sequence detected in *M. rufus* (PP194257) grouped with hemoplasma sequences previously identified in *M. rufus* from Belize and *Tadarida brasiliensis* from the United States.

## 4. Discussion

This study was conducted through a coordinated and systematic effort of field data collection in the Pantanal. Hemoplasmas were detected in bats belonging to four species, namely, *G. soricina* (21MT1C155 and 21MT1C172), *M*. *rufus* (21MT1C289), *D. rotundus* (21MT1C295), and *M. temminckii* (21MT1C122), corresponding to an overall occurrence of 3.2% (5/157) in the sampled population. The hemoplasma-positive specimens were collected in the municipalities of Santo Antônio do Leverger and Poconé, which may be related to the higher number of specimens examined from these areas. The relatively low detection rate observed herein may be influenced by biological and methodological factors, including tissue tropism, bacterial load, stage of infection, and the type of tissue analyzed. Previous studies have also reported hemoplasmas in *G. soricina*, *Molossus* spp., and *D. rotundus* from different regions of Brazil, including the Pantanal biome of Mato Grosso State [9,12,14,26].

Hemoplasma DNA was detected in two members of the family Molossidae, *M. temminckii* and *M. rufus*. Notably, the detection in *M. temminckii* (specimen 21MT1C122; PP193952) represents, to our knowledge, the first molecular evidence of hemotropic mycoplasma infection in this bat species. In the 16S rRNA phylogeny, the sequence obtained from *M. temminckii* clustered with hemoplasmas previously detected in molossid bats, including *M. rufus*, *M. alvarezi*, and *Molossus* sp. from Brazil and Belize. Likewise, the hemoplasma sequence detected in *M. rufus* (specimen 21MT1C289; PP194257) further corroborates previous reports of hemotropic *Mycoplasma* infection in this bat species from Brazil [26]. In the 23S rRNA phylogeny, the sequence obtained in the present study was positioned in close proximity to a cluster comprising hemoplasma sequences previously detected in *M. rufus* from Belize and *T. brasiliensis* from the United States. Notably, all these bat species belong to the family Molossidae, suggesting close phylogenetic relationship among hemoplasma lineages associated with insectivorous molossid bats. In addition to their taxonomic proximity, these species share similar insectivorous feeding habits, which may favor exposure to comparable ecological sources of infection. Although the taxonomic relationships among bat-associated hemoplasmas remain incompletely resolved, the observed phylogenetic relationships support the occurrence of related hemoplasma lineages among insectivorous bats belonging to the family Molossidae. Together, these findings expand the currently known host range of bat-associated hemoplasmas and contribute additional information regarding their genetic diversity and geographic distribution in neotropical bats.

Among the positive bats identified in this study, *G. soricina* deserves particular attention because hemoplasma DNA was characterized using both the 16S and 23S rRNA gene markers. In the 16S rRNA phylogeny, the sequence obtained from *G. soricina* (PX149901), corresponding to specimen 21MT1C172, clustered within a strongly supported clade with another hemotropic mycoplasma sequence (PQ764804) previously detected in *G. soricina* from Brazil. Likewise, the 23S rRNA sequence generated from specimen 21MT1C172 grouped with the sequence obtained from another *G. soricina* specimen detected in the present study (21MT1C155), forming a highly supported cluster composed predominantly of bat-associated hemoplasmas. Notably, these two *G. soricina* specimens were captured approximately 150 km apart (Figure 1; sites 12 and 14), indicating that similar hemoplasma genotypes can be detected in geographically separated populations of this bat species within the Pantanal biome. Previous studies have also reported hemoplasmas in *G. soricina* from other Brazilian regions, including the states of Tocantins and Mato Grosso do Sul [9,26], indicating that hemotropic *Mycoplasma* spp. have been detected in this bat species across distinct ecological settings in Brazil.

The detection of hemoplasma DNA in *D. rotundus* (specimen 21MT1C295; PP197662) is consistent with previous reports identifying this hematophagous bat as a commonly reported host of hemotropic *Mycoplasma* spp. in South America [12,26]. In the 23S rRNA phylogeny, the sequence obtained in the present study clustered with a hemoplasma sequence previously detected in *D. rotundus* from Brazil (OR753267), with strong bootstrap support (100%), indicating a close phylogenetic relationship between these isolates. Similar hemoplasma genotypes have also been reported in vampire bats from Peru and Belize [12]. Collectively, these findings indicate that related hemoplasma genotypes have been detected in geographically distinct populations of *D. rotundus* and further reinforce the frequent association of this bat species with hemotropic *Mycoplasma* spp. in the Neotropics.

Overall, the analyses revealed phylogenetic variation among the hemoplasma sequences detected in bats from the Pantanal. However, the small number of positive specimens, the partial nature of the 16S and 23S rRNA gene fragments, and the limited taxonomic resolution of ribosomal markers preclude definitive taxonomic classification and broader ecological inferences. Future studies employing multilocus approaches or whole-genome sequencing may provide additional insights into the evolutionary relationships and taxonomic status of hemoplasmas circulating in Neotropic bats.

## 5. Conclusions

Our findings expand current knowledge regarding the occurrence of hemotropic *Mycoplasma* spp. in bats from the Pantanal biome. Hemoplasma DNA was detected in four bat species, including the first molecular evidence of hemotropic mycoplasma infection in *M. temminckii*. Phylogenetic analyses demonstrated the association of the detected sequences with previously described bat-associated hemoplasmas. These results contribute to the understanding of hemoplasma occurrence in bats inhabiting one of the world’s largest tropical wetlands.

## Figures and Tables

**Table 1:** Data from bat samples positive for hemotropic *Mycoplasma* spp. detected in the Brazilian Pantanal.

Identification	Municipality	Locality	Family	Species	Sex	Amplified Gene *
21MT1C122	Santo Antônio do Leverger	8	Molossidae	*Molossops temminckii*	ND	16S (PP193952)
21MT1C155	Poconé	14	Phyllostomidae	*Glossophaga soricina*	Female	23S (PP197661)
21MT1C172	Santo Antônio do Leverger	12	Phyllostomidae	*Glossophaga soricina*	Female	16S (PX149901) 23S (PP194256)
21MT1C289	Santo Antônio do Leverger	8	Molossidae	*Molossus rufus*	Female	23S (PP194257)
21MT1C295	Poconé	7	Phyllostomidae	*Desmodus rotundus*	Male	23S (PP197662)

Locality: Locality in Figure 1; ND: Not determined; * Genbank accession number.

## Data Availability

The sequences are available under GenBank accession numbers PP193952, PP197661, PX149901, PP194256, PP194257 and PP197662.

## Supplementary Materials

The following supporting information can be downloaded at: https://www.mdpi.com/article/10.3390/pathogens15060654/s1, Table S1: Geographic coordinates of bat collection sites, their municipalities and localities in the Pantanal and Cerrado biomes, Mato Grosso State, Brazil; Table S2: Sequences retrieved from GenBank; Table S3: BLAST analysis of hemotropic *Mycoplasma* spp. sequences detected in bats from the Pantanal biome, Mato Grosso State, Brazil.
