# Supplementary material for: Molecular Characterization of Hemotropic Mycoplasma spp. From Bats (Chiroptera) in the Northern Pantanal, Brazil
Source: Pathogens. 2026 Jun 22;15(6):654. doi: 10.3390/pathogens15060654 (PMC13305952; doi:10.3390/pathogens15060654)
Supplement: Supplementary file 1 [file pathogens-15-00654-s001.zip › pathogens-4324805-supplementary.pdf]

**Table S1.** Geographic coordinates of bat collection sites, their municipalities and localities in the Pantanal and Cerrado biomes, Mato Grosso State, Brazil.

| Municipality | Location in map* | Geographic Coordinates |
|---|---|---|
| Nobres | 1 Pé-de-serra ranch | −14°37′48″; −056°09′27″ |
| Cuiabá | 2 INPP[a] | −15°36′23″; −056°03′45″ |
| | 3 Urban area NDL[b] | −15°36′36″; −056°07′28″ |
| Poconé | 4 São Pedro ranch | −16°06′55″; −057°03′47″ |
| | 5 Pouso Alegre | −16°30′11″; −056°44′44″ |
| | 6 Pouso Alegre | −16°31'23"; −56° 44' 47" |
| | 7 Rio Claro | −16°37′07″; −056°44′13″ |
| | 14 Porto Cercado | −16°30′43″; −056°24′57″ |
| Santo Antônio do Leverger | 8 Recanto AME+[c] | −15°48′22″; −056°04′53″ |
| | 9 Baía de Sinhá Mariana | −16°20′24″; −055°53′57″ |
| | 10 Mimoso | −16°11′42″; −055°48′25″ |
| | 11 Mimoso | −16°12′40″; −055°48′26″ |
| | 12 Mimoso | −16°14′15″; −055°46′45″ |
| | 13 Baía São João | −16°44′31″; −055°33′05″ |

*Figure 1;

[a] INPP: Instituto Nacional de Pesquisas do Pantanal;

[b] Colégio Notre Dame de Lourdes;

[c] Associação Missão Enchei-vos.

**Table S2.** Sequences retrieved from GenBank.

| Genbank accession number | Location | Organism | Host species | Amplified gene |
|---|---|---|---|---|
| OR753267 | Brazil | uncultured *Mycoplasma* sp. | *Desmodus rotundus* | 23S |
| OQ518944 | Belize | '*Ca.* Mycoplasma haematomolossi' | *Molossus rufus* | 23S |
| OQ359166 | USA | '*Ca.* Mycoplasma haematotraderitadaridae' | *Tadarida brasiliensis* | 23S |
| OR055988 | Belize | '*Ca.* Mycoplasma haematophyllostomi' | *Phyllostomus discolor* | 23S |
| OQ518934 | Belize | uncultured *Mycoplasma* sp. | *Artibeus phaeotis* | 23S |
| OQ518945 | Belize | uncultured *Mycoplasma* sp. | *Desmodus rotundus* | 23S |
| OQ518933 | Belize | uncultured *Mycoplasma* sp. | *Desmodus rotundus* | 23S |
| OL963932 | Thailand | uncultured *Mycoplasma* sp. | *Sus scrofa domesticus* | 23S |
| MN692881 | Brazil | uncultured *Mycoplasma* sp. | *Coendou villosus* | 23S |
| NR076944 | USA | *Mycoplasma haemocanis* | *Canis familiaris* | 23S |
| NR076563 | * | *Ureaplasma parvum* | * | 23S |
| OR016505 | Belize | uncultured *Mycoplasma* sp. | *Molossus alvarezi* | 16S |
| MH245175 | Belize | uncultured *Mycoplasma* sp. | *Molossus rufus* | 16S |
| MK353861 | Belize | uncultured *Mycoplasma* sp. | *Molossus rufus* | 16S |
| MN710412 | Brazil | uncultured *Mycoplasma* sp. | *Molossus sp.* | 16S |
| OQ407836 | USA | '*Ca.* Mycoplasma haematoselmanitadaridae' | *Tadarida brasiliensis* | 16S |
| OQ407847 | USA | uncultured *Mycoplasma* sp. | *Tadarida brasiliensis* | 16S |
| OP795503 | Brazil | uncultured *Mycoplasma* sp. | *Nasua nasua* | 16S |
| KJ530704 | Iran | '*Ca.* Mycoplasma turicense' | *Herpestes javanicus* | 16S |
| KM275265 | Brazil | '*Ca.* Mycoplasma turicense' | *Felis catus* | 16S |
| ON614705 | Somalia | uncultured *Mycoplasma* sp. | *Bos taurus* | 16S |
| EU367965 | Japan | '*Ca.* Mycoplasma haematobovis' | *Bos taurus* | 16S |
| PQ471487 | Brazil | *Mycoplasma haemofelis* | *Leopardus geoffroyi* | 16S |
| KP715860 | Brazil | *Mycoplasma haemocanis* | *Canis familiaris* | 16S |
| KM275242 | Brazil | *Mycoplasma haemofelis* | *Felis catus* | 16S |
| MF377463 | Turkey | *Mycoplasma ovis* | *Ovis aries* | 16S |
| KM275248 | Brazil | '*Ca.* Mycoplasma haematominutum' | *Felis catus* | 16S |
| PV081195 | Iraq | *Ureaplasma parvum* | *Homo sapiens* | 16S |

**Table S3.** BLAST analysis of hemotropic *Mycoplasma* spp. sequences detected in bats from the Pantanal biome, Mato Grosso State, Brazil.

| Sample ID | Host species | Gene | Sequence length | Best BLAST hit | GenBank accession | Identity (%) | Query coverage | E-value | Country of origin |
|---|---|---|---|---|---|---|---|---|---|
| 21MT1C122 | *Molossops temminckii* | 16S | 864 bp | Uncultured *Mycoplasma* sp. clone A3 | MH245175 | 99.03% | 100% | 0.0 | Belize |
| 21MT1C172 | *Glossophaga soricina* | 16S | 628 bp | Uncultured *Mycoplasma* sp. clone 22052 | PQ764804 | 99.68% | 100% | 0.0 | Brazil |
| 21MT1C155 | *Glossophaga soricina* | 23S | 711 bp | Uncultured *Mycoplasma* sp. clone 5 | PV364143 | 99.00% | 100% | 0.0 | Brazil |
| 21MT1C172 | *Glossophaga soricina* | 23S | 728 bp | Uncultured *Mycoplasma* sp. clone 209 | PV364144 | 90.78% | 98% | 0.0 | Brazil |
| 21MT1C289 | *Molossus rufus* | 23S | 587 bp | '*Ca.* M. haematotraderitadaridae clone OK70' | OQ359160 | 99.83% | 100% | 0.0 | USA |
| 21MT1C295 | *Desmodus rotundus* | 23S | 476 bp | Uncultured *Mycoplasma* sp. clone 144 | OR753267 | 99.58% | 100% | 0.0 | Brazil |
